# Cheese and Healthy Diet: Associations With Incident Cardio-Metabolic Diseases and All-Cause Mortality in the General Population

**DOI:** 10.3389/fnut.2019.00185

**Published:** 2019-12-17

**Authors:** Louise H. Dekker, Petra C. Vinke, Ineke J. Riphagen, Isidor Minović, Manfred L. Eggersdorfer, Ellen G. H. M. van den Heuvel, Leon J. Schurgers, Ido P. Kema, Stephan J. L. Bakker, Gerjan Navis

**Affiliations:** ^1^Division of Nephrology, Department of Internal Medicine, University Medical Center Groningen, University of Groningen, Groningen, Netherlands; ^2^Department of Epidemiology, University Medical Center Groningen, University of Groningen, Groningen, Netherlands; ^3^Department of Laboratory Medicine, University Medical Center Groningen, University of Groningen, Groningen, Netherlands; ^4^Friesland Campina, Amersfoort, Netherlands; ^5^Department of Biochemistry, Cardiovascular Research Institute Maastricht, Maastricht University, Maastricht, Netherlands

**Keywords:** nutrition, diet, vitamin K2, cardio-metabolic diseases, all-cause mortality

## Abstract

**Background:** Many countries have established Food-Based Dietary Guidelines (FBDG). For some foods, such as cheese, there is no consensus on whether or not to include them in these guidelines. Cheese may, however, be an excellent source of vitamin K2, which is a macronutrient with demonstrated positive results on cardiovascular-related outcomes.

**Aim:** First, we assessed the role of cheese within the recently developed Lifelines Diet Score (LLDS), a score based on the Dutch FBDG 2015 in relation to incident cardio-metabolic diseases and all-cause mortality. Secondly, we assessed the association of cheese intake with desphospho-uncarboxylated matrix Gla protein (dp-ucMGP), a marker for functional vitamin K2 status, in a subset of the population.

**Methods:** From the Lifelines cohort study, 122,653 adult participants were included to test the association between de LLDS and health outcomes. In a subset of 1,059 participants aged 60–75 years, dp-ucMGP levels were measured. Dietary intake was assessed using a 110-item Food Frequency Questionnaire. Logistic regression were applied, adjusted for relevant confounders.

**Results:** Median cheese intake was 23.5 [12.6–40.6] g/day. We found a positive correlation between cheese intake and the LLDS (Spearman's rho = 0.024, *p* < 0.001). The LLDS in quintiles was associated with T2DM [OR (95% CI) Q5 (healthy diet) vs. Q1 (poor diet) = 0.54 (0.43–0.67)] and all-cause mortality [Q5 vs. Q1 = 0.62 (0.50–0.76)]. Inclusion of cheese did not alter these associations. Additionally, we found no significant association of total cheese intake with plasma dp-ucMGP levels.

**Conclusion:** In this population-based cohort study, the inclusion of cheese in the LLDS did not change the inverse associations with incident cardio-metabolic diseases and all-cause mortality. Furthermore, we found no significant association of total cheese intake with plasma dp-ucMGP. The results suggest that cheese is a neutral food group that fits a healthy diet.

## Introduction

The consumption of a variety of foods is needed to support growth, provide strength, improve cognitive function, and reduce susceptibility to chronic diseases, illnesses, and infection (1). To help address the nutrition concerns of populations, many countries have established Food-Based Dietary Guidelines (FBDG) (2). These FBDGs are expressed in terms of food and diet rather than in nutrients, to be more easily understood and used by community members, and are created to inform the public about consuming a healthy diet (2). Additionally, substantial evidence indicates that foods and dietary patterns have a stronger influence on chronic disease risk than individual nutrients (3, 4).

In the Netherlands, the Dutch Health Council issued their FBDG in 2015 (3). The guidelines are the result of a systematic and critical evaluation of international peer reviewed literature on relations of foods, dietary patterns, and nutrients with causal risk factors and chronic disease risk. Based on the evidence provided by the Dutch Health Council (3), a food group classification has been made, in which groups were categorized as positive, negative, neutral, or unknown, based on the evidence regarding their health effects (5).

Although cheese is sometimes categorized within the food group “dairy,” the composition of cheeses highly differs from dairy products like milk and yogurt, resulting in e.g., differential cardiovascular health effects (6). In the food group classification mentioned above (5), cheese is considered to be a separate food group with unknown health effect. Nevertheless, modest evidence of an association between cheese intake and coronary heart disease and stroke has previously been reported (7). Additionally, among subjects with an impaired glycemic state at baseline, cheese intake was found to be inversely associated with incident diabetes, even after adjusting for BMI and other risk factors (8).

Cheeses are often salted, contributing to high sodium intake (6). Nevertheless, cheese is also nutrient rich, providing a wide range of crucial vitamins (A, B6, B12, D, and K), minerals (calcium, iodine, magnesium, potassium, phosphorus, and zinc), fats, proteins, and other micro constituents (9, 10). Adequate intake of these components is difficult in a diet low in dairy (9). For example, dairy products can provide up to 60% of the recommended daily allowance (RDA) of calcium (10). Additionally, fermented dairy products such as cheese, are an excellent source of fat-soluble vitamins such as vitamin K2 (11–13), vitamin A, and vitamin E (14).

In the Netherlands, cheese is one of the food groups that contribute substantially to the daily food intake (15, 16). In this context it is worthwhile to further study the effect of cheese on health outcomes. The Lifelines cohort, a large general population cohort in the three northern provinces of the Netherlands provides the possibility to assess the role of cheese in a healthy diet in a large representative population. The aim of the present study is two-fold. First, we will assess the possible role of cheese within the recently developed Lifelines Diet Score (LLDS) (5), a score based on the Dutch FBDG 2015. Secondly, we will assess the association between cheese intake and desphospho-uncarboxylated matrix Gla protein (dp-ucMGP), a marker for functional vitamin K2 status (17) in a subset of the population, and is considered a novel risk factor for mortality and CVD (18).

## Methods

### Cohort Design and Study Population

The LifeLines cohort study is a prospective population-based cohort study examining the health and health-related behaviors of 167,729 persons living in the North of the Netherlands. The overall design and rationale of the study have been described in detail elsewhere (19, 20). Participants were included in the study between 2006 and 2013, and written informed consent was obtained from all participants. So far, four assessment rounds took place [T1 = baseline, median + IQR of time in months to follow-up rounds: T2 = 13 [12-14], T3 = 24 [23-27], T4 = 44 [35-51]. The Lifelines study is conducted according to the principles of the Declaration of Helsinki and approved by the Medical Ethics Committee of the University Medical Center Groningen, the Netherlands.

### Lifelines Population

The Lifelines cohort includes 152,662 adults (age 18–93) and dietary information from food frequency questionnaires (FFQ) was available for 144,095 of them. FFQ data was considered unreliable when the ratio between reported energy intake and basal metabolic rate, calculated with the Schofield equation (21), was below 0.50 or above 2.75, or when energy intake was below 800 kcal/day (males) or 500 kcal/day (females). Fourteen thousand seven hundred and thirty-two participants with unreliable dietary intake data were excluded, leaving 129,363 participants in the study. Furthermore, participants who self-reported to have (had) a stroke, myocardial infarction, heart failure, or diabetes [all types, in addition to self-reporting: fasting glucose ≥ 7 mmol/l, HbA1c ≥ 6.5 % or medication use (ATC A10A/A10B)] at baseline, were excluded, leaving 122,653 participants in this study (Figure S1).

### Lifelines Dp-ucMGP Sub-population

Dp-ucMGP measurements were performed in a subset of the LifeLines population consisting of 1,600 subjects, equally distributed over gender and socio-economic status (SES), between 60 and 75 years of age. This subset was selected to investigate vitamin status and subclinical micronutrient deficiency in low vs. high socio-economic status (SES). Since education is more differentiating than income in the Dutch population, classification of SES was based on educational status. Low SES was defined as never been to school or elementary school only, or completed lower vocational or secondary schooling; high SES was defined as completed higher vocational schooling or education. As in the total Lifelines population, participants with missing or unreliable dietary intake data were excluded (*n* = 295) and participants who reported to have (had) a stroke, myocardial infarction, heart failure or diabetes (all types) at baseline (*n* = 201), were excluded. Furthermore, participants reporting to use vitamin K antagonists (*n* = 37) were excluded from this study, leaving 1,059 participants of this sub-cohort in the study.

### Data Collection and Measurements

Self-administered questionnaires were used to collect data regarding demographics (education) and lifestyle (smoking, alcohol, physical activity, diet). The validated short questionnaire to assess health-enhancing physical activity (SQUASH) was used to assess physical activity (22). Leisure time and Commuting Physical activity, including sports, at moderate (4.0–6.4 MET) to vigorous (≥6.5 MET) intensity (LC_MVPA) was calculated in minutes per week (22). Anthropometric measurements and blood pressure were measured by well-trained staff. BMI was calculated as weight (kg) divided by height squared (m^2^).

Blood samples were collected in fasting state between 8.00 and 10.00 a.m. and subsequently transported to the Central Lifelines Laboratory in the University Medical Center Groningen. Functional vitamin K2 status was assessed by measuring dp-ucMGP in EDTA plasma using a dual-antibody enzyme-linked immunoassay [InaKtif MGP (IDS-iSYS) assay]. The lower limit of quantitation of the InaKtif MGP assay was 300 pmol/L. Serum creatinine (SCr) was measured via an enzymatic assay with colorimetric detection on a Roche Modular chemistry analyzer (Roche, Basel, Switzerland). The creatinine-based CKD-EPI formula was used to obtain the estimated glomerular filtration rate (eGFR) (23). Other laboratory measurements were assessed by commercially available assays on a Roche Modular chemistry analyzer (Roche, Basel, Switzerland).

### Dietary Assessment

To assess dietary intake in the LifeLines Cohort, a 110-item semi-quantitative baseline FFQ assessing food intake over the previous month was developed by the Wageningen University using the Dutch FFQTOOL™, in which food items were selected based on the Dutch National Food Consumption Survey of 1997/1998 (24). Energy and macronutrient intake was estimated from the FFQ data by using the Dutch food composition database of 2011 (25). Alcohol consumers were defined as those participants who consumed at least one alcoholic beverage in the past month.

Cheese intake was assessed with three main questions, asking for habitual consumption of cheese on bread, bread-rolls or crackers, with hot meals and as snack. Additionally, it was asked what type of cheese was most frequently chosen (low fat cheese (20/30% fat), regular high fat cheese (40/48% fat), cream cheese or foreign cheeses (e.g., brie or blue cheese). From these data, daily cheese intake in g/day was calculated.

### The Lifelines Diet Score

The Lifelines Diet Score (LLDS) was calculated as a measure of relative diet quality. The development of this food-based diet score has been described in detail elsewhere (5). In short, the LLDS is based on the scientific evidence underlying the 2015 Dutch Dietary Guidelines, and ranks the relative intake of nine food groups with proven positive health effects (vegetables, fruit, whole grain products, legumes and nuts, fish, oils and soft margarines, unsweetened dairy, coffee, and tea) and three food groups with proven negative health effects (red and processed meat, butter and hard margarines and sugar-sweetened beverages) (3). For each of the food groups, quintiles of consumption in grams/1,000 kcal are determined and awarded zero to four points, with four points being awarded to the highest quintile of consumption for positive food groups, and to the lowest quintile for negative food groups. The sum of the 12 component scores resulted in a LLDS score ranging from 0 to 48. The LLDS scores were then categorized into quintiles, with quintile 1 including 20% of participants with the lowest diet quality and quintile 5 including 20% of participants with the highest diet quality. As previously shown, the LLDS is higher in women and positively associated with age category and educational level. For men, mean LLDS ranged from 19.5 (SD = 5.30) in males aged below 40 with low educational level, to 25.9 (SD = 5.50) in highly educated males aged 60 or higher. For women, this range is 20.8 (SD = 5.74) to 29.1 (SD = 5.61) (5).

To investigate the role of cheese in the LLDS, cheese was included in the LLDS as an additional, 13th group. In line with the methods used for other food groups of the LLDS, quintiles were made for cheese intake in grams per 1,000 kcal. Higher scores were either awarded to the quintile of highest intake (when cheese was considered a positive food group), or to the quintile of lowest intake (when cheese was considered a negative food group).

In collaboration with the National Institute for Public Health and the Environment (RIVM), the Netherlands Nutrition Center has calculated various diets that comply with the Dutch dietary guidelines and with the Dietary Reference Values (26). Based on the saturated fat and sodium dietary reference values, a maximum for cheeses of 40 g/day has been set to fit a healthy diet Therefore, a third approach to score cheese intake was used, in which intake up until 40 g was scored as positive, and intake above 40 g as negative. This resulted in four variants of the LLDS to be investigated in statistical analyses.

### Clinical End Points

In the present study, we examined associations of LLDS (with or without cheese) with incidence of cardio-metabolic diseases [i.e., stroke, myocardial infarction, heart failure, and type 2 diabetes (T2DM)] and all-cause mortality. Incident cases of stroke, myocardial infarction and heart failure were based on self-reported questionnaires that were issued in the three follow-up assessment rounds. Incident T2DM was defined as self-reported T2DM according to the questionnaires in the three follow-up rounds, or a fasting glucose ≥7.0 mmol/L or HbA1c ≥ 6.5 mmol/mol during the last follow-up assessment when blood samples were collected. Data on prescribed medication was not available during follow-up. Data on mortality were obtained from the municipal register.

### Statistical Analyses

Continuous data as medians with interquartile ranges (IQR) because of the non-normal distribution of many of the variables involved. Discrete and categorical data are presented as frequencies (%). Logistic regression was applied to investigate the association of cheese intake in quintiles (based on intake in g/1,000 kcal) and incident stroke, myocardial infarction, heart failure, T2DM and all-cause mortality. To evaluate the effect of inclusion of cheese in the LLDS on the association of the LLDS with clinical end points, logistic regression was applied on the four defined LLDS (with or without cheese) and these same five health outcomes. For cardio-metabolic health outcomes, only participants with complete follow-up were included in the analyses since it was unknown whether participants who dropped-out became an incident case or not. For all-cause mortality this does not apply, since this outcome does not rely on self-reporting.

Furthermore, in the subset as described above, we assessed whether cheese intake was associated with dp-ucMGP, a marker for functional vitamin K2 status and a novel risk factor for mortality and CVD (18). The association was investigated using logistic regression analyses with dp-ucMGP dichotomized into ≤ 300 vs. >300 pmol/L as the dependent variable.

All regression analyses were adjusted for relevant confounders including energy intake, education level, age, gender, smoking status, alcohol intake, leisure time, and commuting moderate-vigorous physical activity and BMI. Analyses with quintiles of cheese intake were additionally adjusted for the regular LLDS, and with respect to the dp-ucMGP analysis were additionally adjusted for eGFR. Furthermore, it was tested whether gender was an effect modifier in all regression analyses involving cheese consumption by including the interaction-term of gender and cheese intake in quintiles.

## Results

### Baseline Characteristics

In the present study, we included 122,653 subjects [median age 44 (IQR 35–51) years, 40.8% male] of the adult Lifelines cohort and 1,059 [aged 64 (62–68) years, 48.8% male] of the subset with available dp-ucMGP data. Baseline characteristics of the total study population and for the subpopulation with available dp-ucMGP measurements are presented in Table 1. The median cheese intake was 23.5 g/day in the total study population and 28.8 g/day in the subset population. The mean LLDS was 24 and 27 in the total and subset study population, respectively. We found a significant correlation between daily cheese intake (in g/day) and the LLDS (Spearman's rho = 0.024, *p* < 0.001).

**Table 1:** Baseline characteristics of the total study population and subset with available dp-ucMGP measurements.

	**Total study population**	**Subset with available dp-ucMGP**
	**(*N* = 122,653)**	**(*n* = 1,059)**
**Demographics**		
Male gender (n, %)	50,101 [40.8]	513 [48.4]
Age (years)	44 [35–51]	64 [62–68]
Education		
Low (%)	2.1%	40.2%
Middle (%)	66.6%	-
High (%)	31.3%	59.8%
Smoking status		
Never (%)	47.1%	34.8%
Former (%)	31.9%	54.0%
Current (%)	21.0%	11.2%
BMI [kg/m^2^]	25.3 [23.0–28.0]	25.8 [23.7–28.4]
**Laboratory parameters**		
dp-ucMGP > 300 pmol/L (n, %)	N.A.	243 [22.9%]
Total cholesterol-HDL ratio	3.38 [2.75–4.22]	3.5 [2.9–4.3]
LDL cholesterol	3.2 [2.6–3.8]	3.6 [3.0–4.2]
Triglycerides [mmol/L]	0.96 [0.7–1.36]	1.0 [0.8–1.4]
Serum creatinine [μmol/L]	72 [64–81]	74 [65–85]
eGFR [mL/min/1.73 m^2^]	98 [87–108]	84 [75–91]
**Nutrition**		
Lifelines Diet Score [0–48]	24 [20–28]	27 [23–31]
Energy intake [kcal/day]	1985 [1636–2412]	1862 [1554–2206]
**Cheese intake**		
Total cheese [g/day]	23.5 [12.6–40.6]	28.8 [17.5–44.2]
High fat cheese [g/day]	16.1 [5.9–32.0]	20.9 [ 7.8–39.6]
40+ cheese/cheese spread [g/day]	0 [0–10.7]	0.9 [0–14.2]
48+ cheese/cheese spread [g/day]	0 [0–6.3]	0 [0–7.1]
Cheese with hot meal [g/day]	1.1 [0–3.0]	0.9 [0–2.8]
Cheese as snack [g/day]	2.7 [0–4.6]	2.7 [0.7–5.9]
Cream cheese or foreign cheeses	0 [0–0]	0 [0–0]
[g/day]		
Low fat cheese		
20+/30+ cheese/cheese spread	0 [0–8.9]	0 [0–12.3]
[g/day]		

### Cheese and Incident Stroke, Myocardial Infarction, Heart Failure, T2DM, and All-Cause Mortality

After a median follow-up time of 3.7 [IQR 2.9–4.3] years for cardio-metabolic diseases, 306 (0.38%) incident cases of stroke, 325 (0.40%) incident cases of myocardial infarction, 859 (1.06%) incident cases of heart failure, and 1,099 (1.36%) incident cases of T2DM occurred. After a median follow-up time of 7 years [IQR 6–8] for all-cause mortality, 1,305 subjects (1.06%) had died.

**Figure 1:**
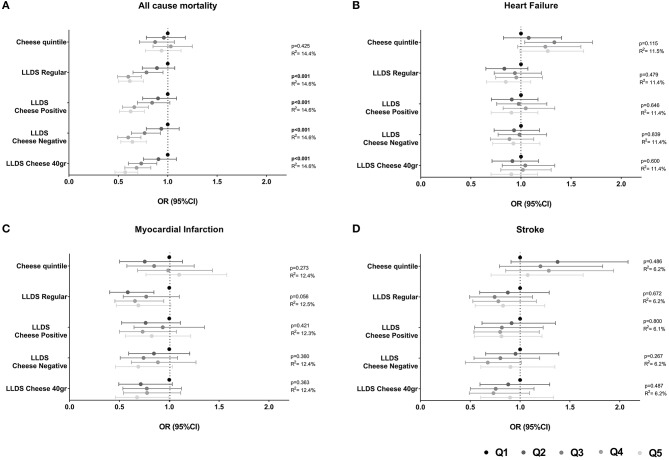
**(A–D)** Associations of quintiles of cheese intake (based on intake in g/1,000 kcal) with cardio-metabolic diseases (*N* = 78,774). Low cheese intake or Q1 of the LLDS as reference. Adjusted for: energy intake, diet quality (LLDS), education level, age, gender, smoking status, alcohol intake, moderate-vigorous physical activity, and BMI.

The association of cheese intake with incident cardio-metabolic diseases is depicted in Table 2 and Figure 1 (upper panel). Cheese intake was not associated with incident cardio-metabolic diseases, adjusted for relevant confounders [OR (95%CI) for Q5 (healthy diet) vs. Q1 (poor diet) = 1.08 (0.71 – 1.63) for stroke, 1.10 (0.77–1.58) for myocardial infarction, 1.26 (0.99–1.62) for heart failure and 1.12 (0.91–1.38) for T2DM]. In addition, the associations of the LLDS with incident cardio-metabolic diseases in which cheese is not included in the score (regular LLDS), as a negative or positive food group, or using a 40 g/day cut-off to define cheese as a positive or negative food group are also shown in Figure 1. In multivariable analysis, higher scores on the regular LLDS were associated with a modest, borderline significant, decrease in the risk for myocardial infarction [OR (95% CI) Q5 vs. Q1 = 0.69 (0.47–1.02), *P* = 0.056], and a substantial and significant decrease in risk for T2DM [0.54 (0.43–0.67), *P* < 0.001] and all-cause mortality [0.62 (0.50–0.76), *P* < 0.001]. The inclusion of cheese either as a positive or negative food group in the LLDS resulted in an attenuation of the borderline association of the LLDS and myocardial infarction, while the inverse associations with T2DM and all-cause mortality remained statistically significant, independent on how cheese was introduced in the score. Inclusion of cheese defined by the 40 g/day cut-off did not affect the associations with cardio-metabolic diseases.

**Table 2:** Associations of quintiles of cheese intake (based on intake in g/1,000 kcal) with cardio-metabolic diseases (*N* = 78,774) and all-cause mortality (*N* = 119,435).

	**Quintiles of Cheese intake**					
**Outcome**	**Q1 (Reference)**	**Q2 [OR (95% CI)]**	**Q3 [OR (95% CI)]**	**Q4 [OR (95% CI)]**	**Q5 [OR (95% CI)]**	***P-value***
Stroke–w/o LLDS	N.A.	1.37 (0.90–2.07)	1.20 (0.79–1.82)	1.28 (0.85–1.93)	1.07 (0.70–1.62)	0.495
Stroke–with LLDS	N.A.	1.38 (0.91–2.08)	1.20 (0.79–1.83)	1.29 (0.86–1.94)	1.08 (0.71–1.63)	0.486
Myocardial infarction–w/o LLDS	N.A.	0.75 (0.50–1.12)	0.84 (0.57–1.23)	0.98 (0.67–1.41)	1.09 (0.76–1.56)	0.267
Myocardial infarction–with LLDS	N.A.	0.76 (0.50–1.13)	0.85 (0.58–1.25)	0.99 (0.68–1.43)	1.10 (0.77–1.58)	0.273
Heart failure–w/o LLDS	N.A.	1.07 (0.82–1.40)	1.34 (1.04–1.72)	1.25 (0.97–1.60)	1.26 (0.99–1.62)	0.115
Heart failure–with LLDS	N.A.	1.07 (0.82–1.40)	1.34 (1.04–1.72)	1.25 (0.97–1.61)	1.27 (0.99–1.63)	0.109
T2DM–w/o LLDS	N.A.	1.13 (0.91–1.40)	1.02 (0.83–1.27)	1.12 (0.91–1.38)	1.08 (0.88–1.33)	0.717
T2DM–with LLDS	N.A.	1.16 (0.94–1.43)	1.05 (0.85–1.31)	1.15 (0.94–1.42)	1.12 (0.91–1.38)	0.595
All-cause mortality–w/o LLDS	N.A.	0.96 (0.78–1.18)	0.87 (0.71–1.07)	1.03 (0.85–1.25)	0.94 (0.78–1.14)	0.425
All-cause mortality–with LLDS	N.A.	0.98 (0.80–1.20)	0.90 (0.73–1.10)	1.06 (0.87–1.28)	0.96 (0.79–1.16)	0.472

*Q1 (low cheese intake) was used as reference quintile*.

*Models are adjusted for: energy intake, education level, age, gender, smoking status, alcohol intake, moderate-vigorous physical activity and BMI, and additionally for diet quality (LLDS)*.

**Figure 2:**
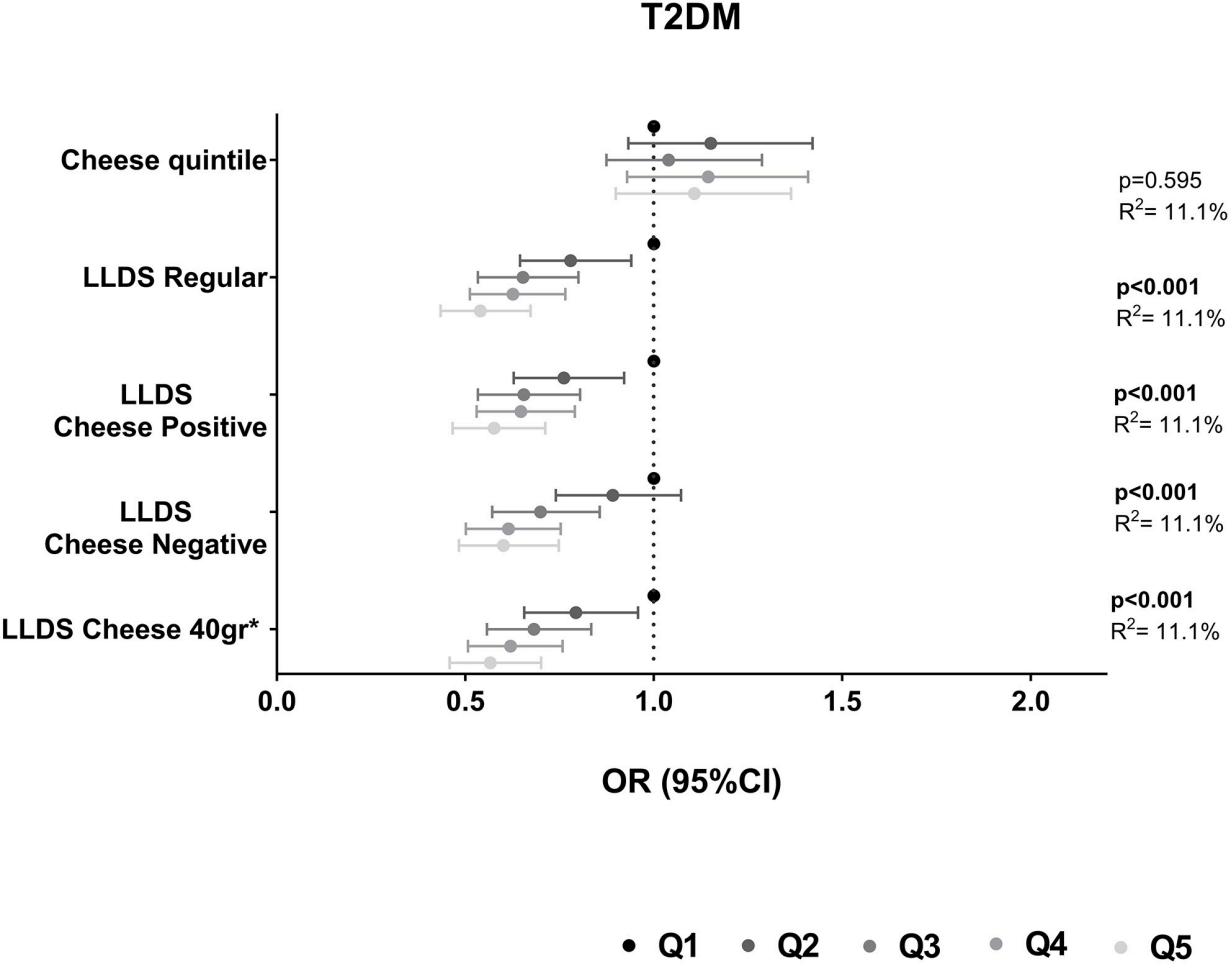
Associations of quintiles of cheese intake (based on intake in g/1,000 kcal) with all-cause mortality (*N* = 119,435). Low cheese intake or Q1 of the LLDS as reference. Adjusted for: energy intake, diet quality (LLDS), education level, age, gender, smoking status, alcohol intake, moderate-vigorous physical activity, and BMI.

The association of cheese and the different LLDS with all-cause mortality is depicted in Figure 2. Adjusted for relevant confounders, cheese was not associated with all-cause mortality [0.94 (0.78–1.14)]. The inclusion of cheese in the LLDS, either as a positive or negative food group, or when applying the 40 g/day cut-off, did not result in a different association with all-cause mortality compared to the regular LLDS. All types of LLDS were significantly inversely associated with all-cause mortality. None of the associations between cheese intake and health were modified by gender.

### Cheese Intake and Dp-ucMGP

The association between cheese intake and dp-ucMGP was assessed in a subset of 1,059 Lifelines participants. A total of 816 participants (77.1%) had a plasma dp-ucMGP level under the detection level of 300 pmol/L. We found no significant association between cheese intake in g/day and plasma dp-ucMGP levels (Spearman's rho = 0.032, *p* = 0.304). However, when investigating the association in 243 participants with dp-ucMGP levels above the detection level of 300 pmol/L, a non-significant but negative association was found (Spearman's rho = −0.029, *p* = 0.656). The association between cheese intake, presented for quintiles of cheese intake, and plasma dp-ucMGP levels is depicted in Table 3. We found no significant trend across these quintiles (*p* for trend = 0.193). Also in confounder adjusted logistic regression, no significant association between cheese intake in quintiles and having a dp-ucMGP above 300 pmol/L was found (*p* = 0.093) (Table 4). Furthermore, the association was not modified by gender.

**Table 3:** Cheese intake (median [range]) and number of participants with dp-ucMGP >300 pmol/L across quintiles of daily cheese intake.

	**Quintiles of cheese intake**					
	**Q1 (*N* = 140)**	**Q2 (*N* = 169)**	**Q3 (*N* = 230)**	**Q4 (*N* = 260)**	**Q5 (*N* = 260)**	***P_**linear trend**_***
**Cheese intake in g/day [median (range)]**	6 (0–10)	16 (10-19)	24 (19-29)	39 (29-44)	64 (45–161)	
**Dp-ucMGP** **>** **300 pmol/L [n (%)]**	23 (16.4%)	44 (26%)	49 (21.3%)	64 (24.6%)	63 (24.2%)	0.193

**Table 4:** Logistic regression analysis to investigate the association of cheese intake quintile (g/day) and the odds of having a dp-ucMGP > 300 pmol/L.

	**Quintiles of Cheese intake**					
	**Q1 (Reference)**	**Q2 [OR (95% CI)]**	**Q3 [OR (95% CI)]**	**Q4 [OR (95% CI)]**	**Q5 [OR (95% CI)]**	***P-value***
Model 1	N.A.	1.92 (1.08–3.40)	1.60 (0.91–2.81)	2.00 (1.15–3.47)	2.11 (1.20–3.73)	0.093
Model 2	N.A.	1.92 (1.08–3.40)	1.60 (0.91–2.82)	2.00 (1.15–3.48)	2.11 (1.20–3.74)	0.093

*N = 1,048*.

*Models 1: Adjusted for energy intake, education level, age, gender, smoking status, alcohol intake, moderate-vigorous physical activity, and BMI. Models 2: adjusted for model 1 + diet quality (LLDS)*.

## Discussion

In this study in the general population, the LLDS was significantly associated with a lower risk of T2DM and all-cause mortality. Inclusion of cheese intake in the LLDS did not alter the associations compared to the regular LLDS. In an elderly subset of the population we investigated whether cheese intake was associated with plasma dp-ucMGP level. We found no significant association of total cheese intake with plasma dp-ucMGP levels.

Our findings are in line with current literature. There is no scientific consensus on the health effects of cheese (27–29). In the present study the absence of an association with health outcomes may be the result of the exclusion of high risk individuals, but may also be explained by the observation that cheese was consumed by those who had a relatively healthy diet (30). However, the association between cheese and health outcomes did not differ in models with and without adjustment of diet quality (LLDS).

The absence of an significant association of cheese with health outcomes may be the result of cheese being a relative “balanced” food with both healthy and less healthy components. While cheese is one of the major sources of saturated fat, which potentially increases plasma levels of low-density lipoprotein-cholesterol (LDL-C) (31), it has also a high content of calcium, which binds with fatty acids in the intestine to form insoluble soap leading to reduced absorption of fat, promoting a higher excretion of fecal fat (32). The both potential positive and negative effects of cheese consumption have been previously been observed with respect to blood lipids (33–35), and may well-explain the largely unchanged associations of the LLDS with and without cheese with clinical end points in the present study.

In the current study, for the regular LLDS we found no association with cardiovascular diseases. This may be due to the relatively low incidence—and hence low discriminative power-in this population with an average age at baseline of 44 years, but also be the result of the exclusively self-reported data that defined these health outcomes. This, in contrast to T2DM and all-cause mortality for which laboratory parameters or municipal register data was available.

Since it has been shown that cheese is the richest source of long-chain menaquinones (vitamin K2) in the Western diet (36), it would be expected that higher cheese intake is associated with lower dp-ucMGP levels. We could, however, not detect such an association in the total sample of participants with measured dp-ucMGP. The absence of this association may be related to the high lower limit of quantification of the dp-ucMGP assay (i.e., 300 pmol/L), as in the small subgroup of participants above this lower limit, an inverse association between cheese intake and dp-ucMGP was found, although not significant. Additionally, it should be noted, however, that actual vitamin K2 content varies considerably and relies on the type of cheese, the time of ripening, the fat content and the region where the cheeses are produced (12). Since the vitamin K2 content varies substantially between different types of cheese, total cheese intake may not be an accurate marker for actual vitamin K2 intake. Given the unavailability of more specific and detailed data on cheese intake (i.e., exact type of cheese and time of ripening) in this study population, we were not able the assess the association of intake of different types of cheese with dp-ucMGP levels or adjust for intake of different types of cheese in analyses. Furthermore, although it has been suggested that vitamin K2 is more effective in activating extra-hepatic vitamin K-dependent proteins than vitamin K1 (16), vitamin K1 also contributes to vitamin K2 status (37). In the present study, intake of vitamin K1 (mainly from green vegetables) may also have contributed to dp-ucMGP levels.

Some limitations of the present study need to be addressed. First, dietary intake was based on self-reported data, and are subject to recall bias. Second, given the lower limit of quantitation of the dp-ucMGP assay (i.e., 300 pmol/L), this assay may not be sensitive enough to detect effects of cheese intake in the LifeLines study population in which 77% of the subjects had dp-ucMGP levels <300 pmol/L. In addition, the cross-sectional design may not have been the most appropriate for testing the association of cheese intake and plasma dp-ucMGP levels, as it is not possible to fully adjust and account for factors affecting plasma dp-ucMGP levels. Intervention studies may be more appropriate to investigate the effect of cheese intake on dp-ucMGP levels. For example, Dalmeijer et al. demonstrated that plasma dp-ucMGP concentrations decreased significantly and dose-dependently after 12 weeks of menaquinone supplementation (38).

Major strengths of the present study are the large sample size, prospective design, long-term follow-up and the availability of data on many potential confounding factors. Furthermore, median daily cheese intake in the dp-ucMGP sub-cohort (28.8 g/day) is highly comparable to the estimated intake in the adult Dutch population (29.0 g/day). As expected, the evidence based LLDS shows clear dose-response associations with health outcomes like T2DM and all-cause mortality, which adds to the validity of the score as a measure of relative diet quality. The absence of a clear (dose-response) association of the LLDS with stroke, myocardial infarction and heart failure may be related to a lower data quality for these cardiovascular health outcomes. In contrary to T2DM and al-cause mortality, the identification of incident cases for these three cardiovascular outcomes relied solely on self-reporting.

In conclusion, in this population based cohort study, the intake of cheese was not associated with cardio-metabolic health outcomes or all-cause mortality and we found no significant association of total cheese intake with plasma dp-ucMGP. Furthermore, the inclusion of cheese in the LLDS did not change the inverse associations with T2DM and all-cause mortality compared to the LLDS without cheese. Therefore, the results suggest that cheese is a neutral food that fits a healthy diet.

## Data Availability Statement

The datasets generated for this study will not be made publicly available. Data is available by contacting the lifelines research office. Please see www.lifelines.nl.

## Ethics Statement

The studies involving human participants were reviewed and approved by The Lifelines study is conducted according to the principles of the Declaration of Helsinki and approved by the Medical Ethics Committee of the University Medical Center Groningen, the Netherlands. The patients/participants provided their written informed consent to participate in this study.

## Author Contributions

LD, PV, IR, GN, and EH contributed to the conception and design of the study. LD, PV, and IR organized the database, performed the statistical analysis, and wrote the first draft of the manuscript. All authors contributed to manuscript revision, read, and approved the submitted version.

### Conflict of Interest

EH was employed by company Friesland Campina. The authors declare that this study received funding from the Dutch Dairy Association. The remaining authors declare that the research was conducted in the absence of any commercial or financial relationships that could be construed as a potential conflict of interest.

## Supplementary Material

Figure S1 Flow-chart of included participants.
