# Supplementary figures and images for: Cheese and Healthy Diet: Associations With Incident Cardio-Metabolic Diseases and All-Cause Mortality in the General Population
Source: Front Nutr. 2019 Dec 17;6:185. doi: 10.3389/fnut.2019.00185 (PMC6927928; doi:10.3389/fnut.2019.00185)

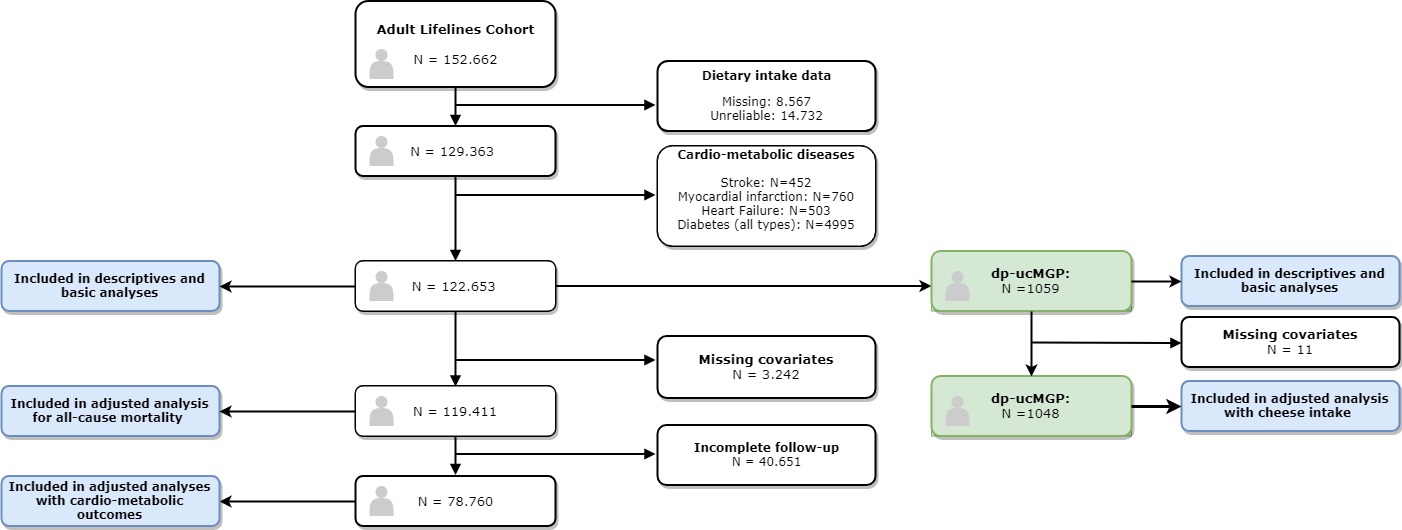

Supplement: Figure S1 — Flow-chart of included participants. [file Image_1.JPEG]
